# Altered expression of hyperpolarization-activated cyclic nucleotide-gated channels and microRNA-1 and -133 in patients with age-associated atrial fibrillation

**DOI:** 10.3892/mmr.2015.3831

**Published:** 2015-05-25

**Authors:** YAO-DONG LI, YI-FAN HONG, YUEERGULI YUSUFUAJI, BAO-PENG TANG, XIAN-HUI ZHOU, GUO-JUN XU, JIN-XIN LI, LIN SUN, JIANG-HUA ZHANG, QIANG XIN, JIAN XIONG, YU-TONG JI, YU ZHANG

**Affiliations:** Department of Cardiology, The First Affiliated Hospital, Xinjiang Medical University, Urumqi, Xinjiang Uyghur 830011, P.R. China

**Keywords:** aged, atrial fibrillation, hyperpolarization-activated cyclic nucleotide-gated, microRNA

## Abstract

Hyperpolarization-activated cyclic nucleotide-gated (HCN) cation channels mediate pacemaker currents in the atrium. The microRNA (miR) families miR-1 and miR-133 regulate the expression of multiple genes involved in myocardial function, including HCN channels. It was hypothesized that age-dependent changes in HCN2, HCN4, miR-1 and miR-133 expression may contribute to age-associated atrial fibrillation, and therefore the correlation between expression levels, among adult (≤65 years) and aged patients (≥65 years), and sinus rhythm was determined. Right atrial appendage samples were collected from 60 patients undergoing coronary artery bypass grafting. Reverse transcription-quantitative polymerase chain reaction (PCR) and western blot analyses were performed in order to determine target RNA and protein expression levels. Compared with aged patients with sinus rhythm, aged patients with atrial fibrillation exhibited significantly higher HCN2 and HCN4 channel mRNA and protein expression levels (P<0.05), but significantly lower expression levels of miR-1 and miR-133 (P<0.05). In addition, aged patients with sinus rhythm exhibited significantly higher expression levels of HCN2 and HCN4 channel mRNA and protein (P<0.05), but significantly lower expression levels of miR-1 and -133 (P<0.05), compared with those of adult patients with sinus rhythm. Expression levels of HCN2 and HCN4 increased with age, and a greater increase was identified in patients with age-associated atrial fibrillation compared with that in those with aged sinus rhythm. These electrophysiological changes may contribute to the induction of ectopic premature beats that trigger atrial fibrillation.

## Introduction

Atrial fibrillation is the most common type of persistent and rapid arrhythmia, with a prevalence of 0.4–1% among the total population (1). The prevalence of atrial fibrillation increases with age, likely due to structural and electrophysiological changes associated with aging and age-associated atrial remodeling. Aging may increase the dispersion of the atrial effective refractory period (2). In addition, L-type Ca channel expression in the right atrium decreases significantly with age, even following the substitution of Ba^2+^ for Ca^2+^ (3). By contrast, the transient outward potassium current (Ito) and persistent potassium current (Isus) increase with age (3). A recent study (4) reported that the funny current (If), mediated by hyperpolarization-activated non-specific cation channels (HCNs), was also elevated in the right atrium of patients with chronic atrial fibrillation.

To the best of our knowledge, to date, no study has investigated whether HCN channels and post-transcription regulators miR-1 and miR-133 contribute to age-associated atrial fibrillation. In the present study, right atrial appendage samples were collected from patients with atrial fibrillation during coronary artery bypass grafting. The expression levels of HCN2 and HCN4 mRNA and proteins, as well as miR-1 and miR-133, among adult and aged patients with sinus rhythm or atrial fibrillation were compared in order to determine whether age-associated changes in expression may contribute to the pathogenesis of age-associated atrial fibrillation.

## Materials and methods

### Patient data

The present study was approved by the ethics committee of the First Affiliated Hospital of Xinjiang Medical University (Urumqui, China). Sixty patients undergoing coronary artery bypass grafting between 2008 and 2013 were enrolled in the study and all provided informed consent. The study population comprised 32 males and 28 females (mean age, 55.12±28.23 years). Patients were divided into three groups according to age and heart rhythm: The aged chronic atrial fibrillation, aged sinus rhythm and adult sinus rhythm groups. Patients in the aged chronic atrial fibrillation group were defined as those ≥65 years, with atrial fibrillation lasting longer than six months as revealed by electrocardiography. Patients with liver and kidney function defects, electrolyte disorders, infections, hyperthyroidism or diabetes were excluded.

### Tissue sample collection and treatment

Baseline clinical data were recorded preoperatively (Table I). *In vitro* circulation was established during surgery, and once the heartbeat stopped, a section of free right atrial appendage, ~1.0×0.5×1.0 cm and weighing ~200 mg, was resected. Following the removal of blood and fat tissue, atrial tissue samples were flash-frozen in liquid nitrogen and stored at −80°C for analysis of target RNA and protein expression.

### RNA extraction and cDNA synthesis

Total RNA was extracted using RNAprep pure tissue kit (Tiangen Biotech Co., Ltd, Beijing, China) according to the manufacturer's instructions. Optical density (OD) values of the RNA extracts were measured at 260 and 280 nm on a UV-spectrophotometer (Thermo Fisher Scientific, San Jose, CA, USA) in order to calculate the purity and concentration. The OD260/OD280 ranged from 1.8–2.0. cDNA was reverse transcribed from mRNA templates using Fermentas reverse transcription kits (Fermentas; Thermo Fisher Scientific, Pittsburgh, PA, USA). Briefly, a 0.1 ng-5 *µ*g total RNA sample and 1 *µ*l oligo (dT) 18 primer were mixed and the volume adjusted to 12 *µ*l with double distilled water (ddH_2_0). The mixture was centrifuged, placed in a 65°C water bath for 5 min and then immediately placed on ice. Subsequently, 4 *µ*l buffer solution, 1 *µ*l RNase inhibitor, 2 *µ*l 10 mM deoxyribonucleotide triphosphate (dNTP) mix 2 and 1 *µ*l M-MLV reverse transcriptase (200 U/*µ*l) were added and this mixture was centrifuged and incubated at 42°C in a water bath for 60 min. The reaction was stopped by heating to 70°C for 5 min. The obtained cDNA was stored at −20°C for subsequent reverse transcription-quantitative PCR (qPCR) analyses.

### Primer sequences

Primers for qPCR anlysis of HCN2 and HCN4 (Table II), and miR-1 and miR-133 (Table III) were designed using Primer Premier 5.0™ software (Premier Biosoft, Palo Alto, CA, USA) based on sequences in GenBank (www.ncbi.nlm.nih.gov/genbank).

### PCR

The PCR reaction system comprised 1 *µ*l sample cDNA, 10 *µ*M forward and reverse primers (0.5 *µ*l for each), 0.15 *µ*l *Taq* enzyme, 2.5 *µ*l buffer and 0.5 *µ*l dNTP, adjusted to a total volume of 25 *µ*l with ddH_2_0. The reaction conditions were as follows: Pre-denaturation at 95°C for 3 min, 30 cycles of denaturation at 94°C for 30 sec, annealing at 56°C for 30 sec, extension at 72°C and a final extension at 72°C for 10 min. PCR products were detected by 1% agarose gel electrophoresis (5 V/m). A Bio-Rad gel imaging system (Bio-Rad Laboratories, Inc., Hercules, CA, USA) was employed for band visualization and gel photography.

### qPCR

qPCR analysis was performed at a series of cDNA dilutions, including 10, 10^2^, 10^3^, 10^4^, 10^5^ and 10^6^. The primers (10 *µ*M) were used at gradient concentrations of 0.25, 0.5, 0.75, 1, 1.25 and 1.5 *µ*l. The amplification efficiency of each primer pair ranged from 0.9–1.1. The primer concentration with the highest amplification efficiency was selected for subsequent quantitative analysis. The qPCR reaction system included 2X of 10 *µ*l SYBR Premix (Tiangen Biotech Co., Ltd), 10 *µ*M forward and reverse primers (0.6 *µ*l of each) and 1 *µ*l cDNA, with total volume adjusted to 20 *µ*l using ddH_2_0. The prepared reaction solution was analyzed using Bio-Rad fluorescence PCR (Bio-Rad Laboratories, Inc.). Reaction conditions were as follows: Pre-denaturation at 95°C for 15 min and 40 cycles of denaturation at 95°C for 10 sec, annealing at 60°C for 10 sec, and extension at 72°C for 20 sec. The fluorescent signal was recorded during the extension phase of each cycle using the CFX96 real-time PCR detection system (Bio-Rad PCR; Bio-Rad Laboratories, Inc.). Melting curve analysis (95–65°C) was performed following the reaction.

### Western blot analysis

Total protein was extracted from tissue lysates for western blot analysis (Beyotime Institute of Biotechnology, Haimen, China). Total protein concentration was determined using bicinchoninic acid reagent kits (Beyotime Institue of Biotechnology). For electrophoretic separation, 20 *µ*g protein per gel lane was boiled in loading buffer and loaded onto 12% polyacrylamide gels. Separated proteins were transferred electrophoretically onto nitrocellulose membranes (Bio-Rad Laboratories, Inc.) and blocked in 5% fat-free powdered milk at 4°C overnight. Following blocking, primary polyclonal rabbit antibodies against HCN2, (cat. no. BS3372; 1:500, Bioworld Technology, Inc., St. Louis Park,. MN, USA) and HCN4 (cat. no. BS3687; 1:250, Bioworld Technology, Inc.), and primary mouse monoclonal antibody against GAPDH (cat. no. sc-365062; 1:500, Santa Cruz Biotechnology Inc., Dallas, TX, USA) were added drop-wise and the membranes were incubated for 2 h at room temperature. Following three washes in Tris-buffered saline and Tween-20 (TBST; 10 min/wash), the corresponding secondary antibody solution was added and membranes were incubated for 1 h at room temperature. Following three additional washes in TBST (Beyotime Institue of Biotechnology), immunolabeling was visualized by electrochemiluminescence (ECL; EMD Millipore, Billerica, MA, USA) and the chemiluminescence signal captured by an imaging system (ChemiDoc^®^-It HR 410 imaging system; UVP, LLC, Upland, CA, USA).

### Statistical analysis

Values are expressed as the mean ± standard deviation or as rate and percentage. All data were analyzed using SPSS 17.0 statistical software (SPSS, Inc., Chicago, IL, USA). Group means of continuous data were compared by one-way analysis of variance and categorical rates and percentages by χ^2^ tests. P<0.05 was considered to indicate a statistically significant difference between values.

## Results

### Patient baseline data

No statistically significant differences in gender ratio or inner diameter of the right atrium were identified amongst the three groups (P>0.05). The aged atrial fibrillation and aged sinus rhythm groups did not differ significantly in mean age or left ventricular ejection fraction (P>0.05), whereas a significant difference in the inner diameter of the left atrium was detected between the two groups (P<0.05). Significant differences in left ventricular ejection fraction (P<0.05), but not the inner diameter of the left atrium (P>0.05), were detected between the aged and adult sinus rhythm groups. Baseline data are summarized in Table I.

### Expression levels of HCN2 and HCN4 channel mRNA in the right atrial appendage vary between groups

**Figure 1:**
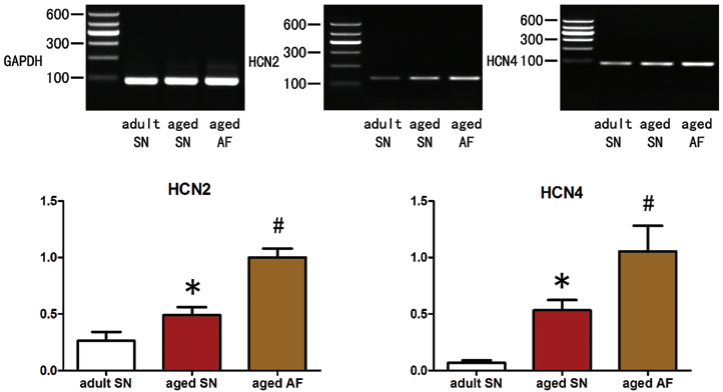
Expression levels of HCN2 and HCN4 channel mRNA. Expression levels of HCN2 and HCN4 channel mRNA as measured by reverse transcription-quantitative polymerase chain reaction in the adult sinus rhythm, aged sinus rhythm and aged atrial fibrillation groups.^*^P<0.05, vs. adult SN group; ^#^P<0.05, vs. aged SN group. SN, sinus rhythm; AF, atrial fibrillation.

Compared with the adult sinus rhythm group, the aged sinus rhythm group exhibited significantly elevated expression levels of HCN2 and HCN4 mRNA (P<0.05; HCN2, 0.49±0.07 vs. 0.26±0.08; HCN4, 0.53±0.09 vs. 0.07±0.02; Fig. 1). Compared with the aged sinus rhythm group, the aged atrial fibrillation group also exhibited significantly enhanced levels of HCN2 and HCN4 mRNA expression (P<0.05; HCN2, 1.00±0.08 vs. 0.49±0.07; HCN4, 1.05±0.23 vs. 0.53±0.09; Fig. 1).

### Expression levels of HCN2 and HCN4 channel proteins in the right atrial appendage vary between groups

**Figure 2:**
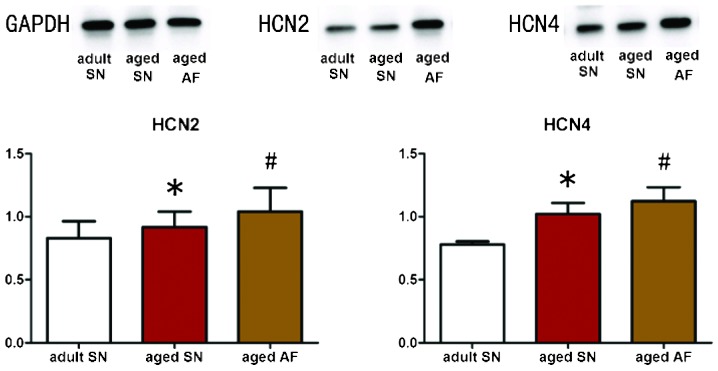
Expression levels of HCN2 and HCN4 channel proteins. Expression levels of HCN2 and HCN4 channel proteins as measured by western blot analysis in the adult sinus rhythm, aged sinus rhythm and aged atrial fibrillation groups.^*^P<0.05, vs. adult SN group; ^#^P<0.05, vs. aged SN group. SN, sinus rhythm; AF, atrial fibrillation.

In accordance with the results of RT-qPCR analysis, the aged sinus rhythm group was demonstrated to have significantly elevated expression levels of HCN2 and HCN4 proteins compared with those of the adult sinus rhythm group (P<0.05; HCN2, 0.92±0.12 vs. 0.83±0.13; HCN4, 1.02±0.08 vs. 0.78±0.02; Fig. 2). Similarly, the aged atrial fibrillation group exhibited significantly enhanced HCN2 and HCN4 protein expression levels compared to those of the aged sinus rhythm group (P<0.05; HCN2, 1.04±0.19 vs. 0.92±0.12; HCN4, 1.12±0.11 vs. 1.02±0.08; Fig. 2).

### Expression levels of miR-1 and miR-133 in the right atrial appendage vary between groups

**Figure 3:**
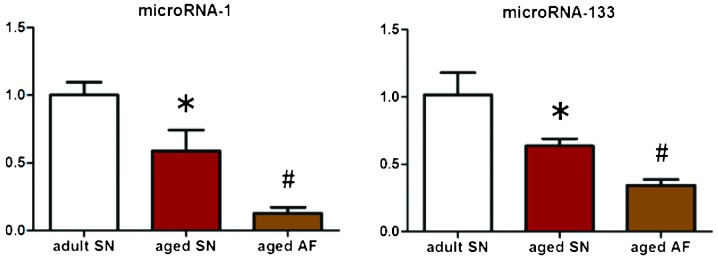
Expression levels of microRNA-1 and -133 in the adult sinus rhythm, aged sinus rhythm and aged atrial fibrillation groups. ^*^P<0.05, aged sinus rhythm group vs. adult SN group; ^#^P<0.05, aged AF group vs. aged sinus rhythm group. SN, sinus rhythm; AF, atrial fibrillation.

Patients in the aged sinus rhythm group exhibited significantly lower expression levels of miR-1 and miR-133 compared to those of the adult sinus rhythm group (P<0.05; miRNA-1, 0.59±0.16 vs. 1.00±0.09; miRNA-133, 0.64±0.05 vs. 1.01±0.17; Fig. 3). Compared with those of the aged sinus rhythm group, the aged atrial fibrillation group also exhibited significantly lower expression levels of miRNA-1 and -133 (P<0.05; miRNA-1, 0.13±0.04 vs. 0.59±0.16; miRNA-133, 0.34±0.04 vs. 0.64±0.05; Fig. 3).

## Discussion

The results of the present study suggested that the mRNA and protein expression levels of HCN2 and HCN4 channels increased in the right atrial appendage with age, whereas the expression levels of post-transcriptional regulators miR-1 and miR-133 declined with age. These age-associated alterations in expression were even more pronounced in aged atrial fibrillation patients compared with those of aged sinus rhythm patients, implicating elevated HCN activity and reduced miR-1/133-mediated regulation of HCN expression in the pathogenesis of atrial fibrillation.

Aberrant changes in pacemaker currents contribute to the generation of rapid arrhythmias. The HCN channels conduct a mixed K^+^/Na^+^ depolarizing current (If), which is activated by hyperpolarization and cyclic adenosine monophosphate (5). The activation of HCN channels contributes towards membrane depolarization during the myocardial diastolic period (6). Despite the vital role of If in cardiac pacemaker activity, the molecular structure of the HCN channels underlying this activity was only relatively recently elucidated (7). Early studies suggested that the expression levels of HCN channel proteins were low in normal myocardial cells outside of the sinoatrial node (8); however, this hypothesis was challenged by subsequent studies. Porciatti *et al* (9) successfully recorded If currents in the human right atrium using whole cell patch-clamping, and Zorn-Paulya *et al* (10) confirmed the existence of HCNs in the left atrium. The kinetics of If differed between the left auricula and left atrial wall, suggesting regional heterogeneity of HCN subtype distribution. Zorn-Paulya *et al* (10) detected HCN2 and HCN4 expression in the human left atrial appendage muscle, which produced an If conferring strong pacemaker cell characteristics to left atrial myocytes. The If, and the associated HCN channels, is also closely associated with arrhythmia (11). A variety of pathological processes lead to abnormally enhanced myocardial HCN channel expression and If augmentation in the atrium or ventricle (12,13). These elevated expression levels may enhance the autorhythmia of myocytes and strengthen ectopic premature beats. Kuwahara *et al* (14) revealed that the transcriptional inhibitor neuron-restrictive silencer factor (NRSF) acted as a regulator of fetal cardiac gene expression. A dnNRSF-expressing transgenic mouse model with gradually progressive myocardial disease was demonstrated to overexpress HCN2 and HCN4 in the ventricle (15). These mice died of sudden arrhythmia aged eight weeks, indicating that HCN2 and HCN4 overexpression in the ventricles may contribute to ventricular arrhythmias (15). Zicha *et al* (12) analyzed atrial myocytes in a canine model of rapid ventricular pacemaker activity and demonstrated that If enhancement, and the associated HCN channel overexpression, contributed to heart failure-induced ventricular arrhythmia.

The pacemaker currents likely participate in the pathogenesis of age-associated atrial fibrillation. The HCN-mediated If current is expressed at high levels in working myocytes exhibiting ectopic premature beats (16). In the present study, whether these channels also contribute to age-associated atrial fibrillation was evaluated. Stillitano *et al* (4) compared right atrial HCN expression between patients that had undergone bypass surgery with persistent atrial fibrillation or sinus rhythm and found that HCN expression was significantly higher in patients with persistent atrial fibrillation, while levels of miR-1 were lower. The prevalence of atrial fibrillation was positively correlated with age (1). Similarly, the prevalence of sinoatrial node disorder (SND) increases with age (17), and SND is frequently accompanied by atrial fibrillation and rapid arrhythmias. Age-associated degeneration of the sinoatrial node also contributes to atrial electrical remodeling (18). Therefore, atrial electrophysiological characteristics and ion channel expression patterns are altered with age (2,3,19,20). Consequently, it was hypothesized that age-associated atrial fibrillation may be correlated with age-associated sinoatrial node degeneration; in addition, it was speculated that with age, atrial and pulmonary HCN channel expression levels may be elevated and the If current may be enhanced, which would lead to an increase in ectopic autorhythmia and may trigger atrial fibrillation. Li *et al* (21) established canine models with age-associated atrial fibrillation or age-associated sinus rhythm, and demonstrated that the If current and HCN4 mRNA expression levels were significantly higher in the age-associated sinus rhythm model, indicating that HCN and the HCN-mediated If, particularly the HCN4 channel current component, may be involved in the pathogenesis of age-associated atrial fibrillation.

In the present study, it was demonstrated that the expression levels of HCN2 and HCN4 mRNA and protein were enhanced in the right atrial appendage of aged sinus rhythm patients compared to those of adult sinus rhythm patients, confirming the presence of the hypothesized age-associated elevation in HCN expression. Furthermore, aged atrial fibrillation patients were found to exhibit higher HCN mRNA and protein expression levels than those of aged sinus rhythm patients. Due to ethical constraints, samples were only collected from the right atrial appendage; however, it was speculated that HCN channel and If current densities may be elevated at other sites of the atrium and in the pulmonary vein. The enhanced expression of atrial HCN and the strengthened If current may result in ventricular premature beats or atrial tachycardia; therefore inducing atrial fibrillation.

HCN channels are regulated by miRs. miRs inhibit the translation of target genes by binding to the complementary sequence of the 3′ untranslated region or by directly modulating mRNA degradation (22). Girmatsion *et al* (23) and Stillitano *et al* (4) demonstrated that the expression of miR-1 was downregulated in patients with persistent atrial fibrillation. miR-1 and -133 are dually regulated in muscle (24), and their expression levels are correlated with the expression of HCN2 and HCN4 channel proteins (25,26). miR-1 and -133 exert inhibitory effects upon HCN2, while miR-1 is also able to downregulate HCN4 expression. Therefore, the overexpression of exogenous miR-1 and miR-133 is able to suppress HCN2 and HCN4 expression (26). Preliminary canine studies by our group (27), revealed that miR-1 and miR-133 expression altered during aging; revealing that expression levels were significantly lower in the aged canine atrium than those in the adult canine atrium. In the present study, the expression levels of miR-1 and miR-133 were found to be lower in the right atrial appendage of aged sinoatrial fibrillation patients than those of the adult sinus rhythm patients, and lower still in adult sinus rhythm patients. This negative correlation between miR-1/miR-133 and HCN2/HCN4 suggested that the downregulation of miR-1 and miR-133 contributed to HCN2 and HCN4 upregulation during aging.

The results of the present study demonstrated a potential role for miR-1 and miR-133 in age-associated HCN channel upregulation in the human atrium. In patients with aged atrial fibrillation, the expression of HCN2 and HCN4 channels was enhanced compared with aged and adult sinoatrial fibrillation patients, whereas expression levels of miR-1 and miR-133 were lower. It was therefore suggested that this age-associated increase in HCN2 and HCN4 expression enhanced the If current and therefore may increase the incidence of ventricular premature beats and atrial tachycardia, triggering atrial fibrillation. Furthermore, these changes in HCN channel and miR expression may be associated with degeneration of the sinoatrial node. Ivabradine, a specific inhibitor of If, is able to reduce the frequency of spontaneous action potentials mediated by the pulmonary vein If current (28). Multiple animal experiments have confirmed that ivabradine is also capable of decreasing ventricular arrhythmias. However, whether ivabradine may represent an effective treatment for age-associated atrial fibrillation remains to be elucidated. Relevant animal experiments are required in order to conclude whether ivabradine may represent a potential therapeutic. These findings therefore improve current knowledge of the association between HCN channels and age-associated atrial fibrillation.

In the present study, only right atrial appendage samples were collected, and therefore whether these changes in expression continue throughout the atrium and pulmonary vein remains unknown. Furthermore, channel expression was investigated at the mRNA and protein levels but the effects of these changes on If current magnitude and kinetics was not investigated. Finally, sinoatrial node function was not evaluated in these patients; therefore, the potential contribution of SND or age-associated sinoatrial node degeneration to these expression changes and clinical conditions were not able to be assessed.

## Figures and Tables

**Table I:** Baseline patient data.

Characteristic	Adult SN (n=20)	Aged SN (n=22)	Aged AF (n=18)
Gender (n)			
Male	14 (70%)	16 (73%)	13 (72%)
Female	6 (30%)	6 (27%)	5 (28%)
Age (years)	40.14±8.73^a^	68.34±5.14	69.13±4.16
LA (mm)	35.03±8.23	36.11±4.22	41.43±6.25
RA (mm)	33.14±3.32	35.32±6.54	36.23±8.33
EF (%)	60.61±3.19^a^	41.27±10.75	41.34±7.29

a P<0.05, adult SN vs. aged SN. LA, inner diameter of the left atrium; RA, inner diameter of the right atrium; EF, left ventricular ejection fraction; adult SN, adult sinus rhythm group; aged SN, aged sinus rhythm group; aged AF, aged atrial fibrillation group.

**Table II:** Sequence of HCN2 and HCN4 primers.

Gene	Sequence	Length (bp)
GAPDH	5′-TGCACCACCAACTGCTTAGC-3′	87
	5′-GGCATGGACTGTGGTCATGAG-3′	
HCN2	5′-CCAGCTGTAAGACAGGGACG-3′	130
	5′-GCGGGCCAAGTATTGCACTT-3′	
HCN4	5′-GGGGAATTCGCAACTGAAGC-3′	83
	5′-TGCTGCGCCCTTAAATCTCT-3′	

HCN, hyperpolarization-activated cyclic nucleotide-gated.

**Table III:** Sequence of the microRNA-1 and -133 primers.

Gene	Sequence
U6	All-in-one™ miRNA qPCR (internal primer) reverse primer provided by the reagent kits
microRNA-1	All-in-one™ miRNA qPCR primer reverse primer provided by the reagent kits
microRNA-133	All-in-one™ miRNA qPCR primer reverse primer provided by the reagent kits

miRNA, microRNA; qPCR, quantitative polymerase chain reaction.
